# Compatibility Studies of Sildenafil-HPBCD Inclusion Complex with Pharmaceutical Excipients

**DOI:** 10.3390/pharmaceutics17091114

**Published:** 2025-08-27

**Authors:** Răzvan Adrian Bertici, Amalia Ridichie, Nicoleta Sorina Bertici, Adriana Ledeţi, Ionuţ Ledeţi, Renata-Maria Văruţ, Laura Sbârcea, Paul Albu, Matilda Rădulescu, Gerlinde Rusu, Dragoș Cătălin Jianu, Ovidiu Fira-Mladinescu

**Affiliations:** 1 Doctoral School, “Victor Babeş” University of Medicine and Pharmacy Timișoara, Eftimie Murgu Square No. 2, 300041 Timișoara, Romania; razvan.bertici@umft.ro; 2 Center for Research and Innovation in Personalised Medicine of Respiratory Diseases, Pulmonology University Clinic, “Victor Babeș” University of Medicine and Pharmacy Timișoara, Eftimie Murgu Square No. 2, 300041 Timișoara, Romania; bertici.nicoleta@umft.ro (N.S.B.); mladinescu@umft.ro (O.F.-M.); 3 Department of Analytical Chemistry, Faculty of Pharmacy, “Victor Babeș” University of Medicine and Pharmacy Timișoara, Eftimie Murgu Square No. 2, 300041 Timișoara, Romania; afulias@umft.ro; 4 Advanced Instrumental Screening Center, Faculty of Pharmacy, “Victor Babeş” University of Medicine and Pharmacy Timișoara, Eftimie Murgu Square No. 2, 300041 Timișoara, Romania; ionut.ledeti@umft.ro (I.L.); sbarcea.laura@umft.ro (L.S.); 5 Pulmonary Hypertension Treatment Unit, Center of Expertise for Adult Rare Respiratory Diseases, Clinical Hospital of Infectious Diseases and Pneumophthisiology “Dr. Victor Babes”, 13 Gheorghe Adam Str., 300310 Timișoara, Romania; 6 Department of Physical Chemistry, Faculty of Pharmacy, “Victor Babeș” University of Medicine and Pharmacy Timișoara, Eftimie Murgu Square No. 2, 300041 Timișoara, Romania; 7 Faculty of Pharmacy, University of Medicine and Pharmacy Craiova, 2-4 Petru Rares Str., 200349 Craiova, Romania; 8 Department of Drug Analysis, Faculty of Pharmacy, “Victor Babeș” University of Medicine and Pharmacy Timișoara, Eftimie Murgu Square No. 2, 300041 Timișoara, Romania; 9 Department of Pharmaceutical Sciences, Faculty of Pharmacy, “Vasile Goldiș” Western University of Arad, 86 L. Rebreanu Str., 310414 Arad, Romania; albu.paul@uvvg.ro; 10 National Institute for Economic Research “Center for Mountain Economy (CE-MONT), Costin C. Kiritescu” of the Romanian Academy, 49 Petreni St., 725700 Vatra Dornei, Romania; 11 Department of Microbiology, Division of Microbiology, “Victor Babeș” University of Medicine and Pharmacy Timișoara, Eftimie Murgu Square No. 2, 300041 Timișoara, Romania; radulescu.matilda@umft.ro; 12 Faculty of Chemical Engineering Biotechnologies and Environmental Protection, University Politehnica Timisoara, 6 Vasile Parvan Boulevard, 300223 Timișoara, Romania; gerlinde.rusu@upt.ro; 13 Department of Neurosciences, First Division of Neurology, “Victor Babeș” University of Medicine and Pharmacy Timișoara, Eftimie Murgu Square No. 2, 300041 Timișoara, Romania; jianu.dragos@umft.ro; 14 Advanced Centre for Cognitive Research in Neuropsychiatric Pathology (NeuroPsy-Cog), Department of Neurosciences-VIII, “Victor Babes” University of Medicine and Pharmacy Timișoara, 156 L. Rebreanu Ave., 300736 Timișoara, Romania

**Keywords:** sildenafil citrate, inclusion complex, cyclodextrin, compatibility study

## Abstract

**Background/Objectives:** In the past two decades, the primary therapeutic use of sildenafil has shifted significantly, from the treatment of angina to managing erectile dysfunction, and since the early 2000s it has been used in the treatment of pulmonary hypertension, particularly pulmonary arterial hypertension. Sildenafil is used as a citrate salt; after oral administration, it presents an absorption of ~90% and an absolute bioavailability of 38%, due to the first-pass effect, such that it belongs to class II of the Biopharmaceutics Classification System. Currently, studies are seeking to obtain new pharmaceutical formulations with an optimized biopharmaceutical profile. In this study, an inclusion complex of sildenafil citrate and 2-hydroxypropyl-beta-cyclodextrin in a molar ratio of 1:1 was obtained and its pharmaceutical compatibility with six pharmaceutical excipients was assessed. For three of these excipients, the presence of chemical interactions with sildenafil citrate has been presented in the literature, and for the other three, compatibility has not been evaluated. **Methods:** To certify the stoichiometry of the obtained inclusion complex molecular modeling, Job’s method and the Benesi–Hildebrand method were employed. Furthermore, we have described the inclusion complex and the obtained binary mixtures via ATR-FTIR and thermal (TG/DTG and DSC) analysis. **Results:** The results indicated a lack of chemical interactions between the inclusion complex and the six pharmaceutical excipients at ambient temperature (confirmed by ATR–FTIR investigations) and the presence of chemical interactions between the inclusion complex and three of the excipients when the mixture was heated under non-isothermal conditions (TG/DTG and DSC investigations). **Conclusions:** This study describes the inclusion complex between sildenafil citrate and 2-hydroxypropyl-beta-cyclodextrin in a molar ratio of 1:1 and its compatibility with several pharmaceutical excipients, results with further applications in the preformulation stage of novel delivery systems.

## 1. Introduction

Over the past two decades, the primary therapeutic use of sildenafil has shifted significantly. Originally used for the treatment of angina, it gained widespread recognition for its effectiveness in managing erectile dysfunction. However, since the early 2000s, sildenafil has increasingly been employed in the treatment of pulmonary hypertension, particularly pulmonary arterial hypertension (PAH) [1].

PAH is a rare condition characterized by a sustained elevation in mean pulmonary arterial pressure (mPAP) above 20 mmHg, confirmed by right heart catheterization [2]. The therapeutic indication for sildenafil in PAH emerged following a series of randomized, controlled, multinational trial studies conducted by leading expert centers [3,4,5,6]. These findings were subsequently endorsed by international organizations such as the European Society of Cardiology (ESC) and the European Respiratory Society (ERS), and the drug was approved for PAH treatment by the U.S. Food and Drug Administration (FDA) and other regulatory agencies [7,8].

Sildenafil was selected for PAH management due to its relatively low risk profile, accessibility and good overall response [9,10]. Its mechanism of action involves the selective inhibition of phosphodiesterase type 5, an enzyme abundantly expressed in pulmonary vasculature, which helps reduce pulmonary vascular resistance and improve hemodynamics [11].

Beyond its cardiovascular applications, sildenafil is currently being investigated for potential neurological benefits. Ongoing studies are exploring its role in enhancing cerebrovascular reactivity, promoting neurogenesis and supporting functional recovery in patients who have experienced stroke [12,13,14,15].

**Figure 1:**
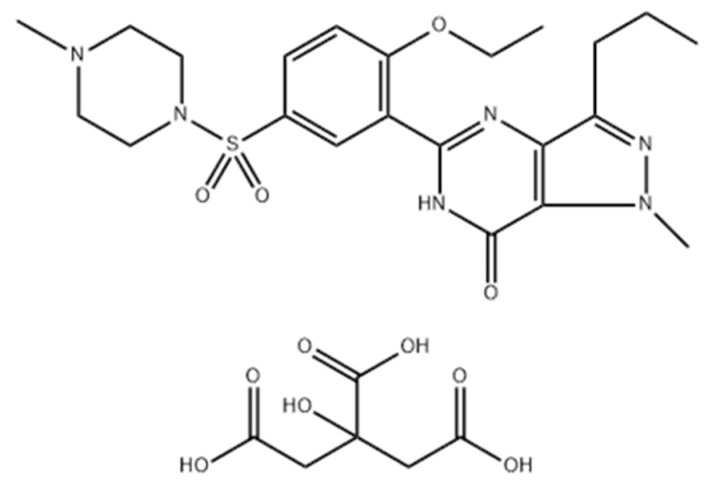
Chemical structure of SC.

Sildenafil is used as a citrate salt (sildenafil citrate, abbreviated SC; chemical structure presented in Figure 1), and regarding its pharmacokinetic profile, it was observed that it suffers an intense hepatic first-pass effect due to the CYP3A4 and CYP2C9 citoenzymes. The metabolites form mainly through N-demethylation of the piperazine and pyrazole rings, N-oxidation and aliphatic dehydroxylation. After oral administration, even if the absorption was ~90%, due to the first-pass effect, the absolute bioavailability was reduced to 38% [16,17]. After metabolization, a metabolite with similar affinity to the phosphodiesterase type 5, named UK-103,320, was observed [16]. SC is mostly eliminated in feces (80%), with 13% eliminated in urine. It presents a low biodisponibility, belonging to class II of the Biopharmaceutics Classification System [18], and limited pharmaceutical formulations are used in the treatment of PAH. Taking these aspects into account, it is necessary to develop new formulations with an enhanced biopharmaceutical profile.

In recent years, research has focused on developing novel pharmaceutical formulations for the administration of SC in PAH, e.g., inhaled sildenafil-loaded PLGA microparticles [19], inhalable SC spray-dried microparticles [20], microneedle-mediated transdermal delivery of SC [21] and bilosomal nanogel [22]. In addition, to improve the biopharmaceutical profile of SC, several inclusion complexes of SC and different CDs in a molar ratio of 1:1 have been obtained (α-CD, β-CD, hydroxypropyl-β-CD, hydroxybutyl-β-CD and γ-CD) [23,24,25,26,27].

### 1.1. Current Level of Knowledge Regarding the ICs of SC

The first study of an inclusion complex containing SC was reported in 2006 by Omari et al., which demonstrated the efficiency of the encapsulation of the active pharmaceutical ingredient with four different cyclodextrins (α-CD, β-CD, hydroxypropyl-β-CD and γ-CD) through phase solubility studies in 0.05 M citrate buffer. The results indicated that the complex formation constant and the increase in the solubility profile of SC varies in the following order: β-CD > hydroxypropyl-β-CD > γ-CD and α-CD, suggesting a better fit of SC in the CD cavity for the β-CD derivatives [26]. In 2010, the first patent of an inclusion complex containing SC and α-CD with therapeutic indications in PAH and erectile dysfunction was reported. The patent shows the variation in the solubility of SC entrapped in α-CD and hydroxypropyl-β-CD with pH level. Although at most pH values the higher solubility of SC is observed for α-CD, at a pH value equal to 7 a higher solubility is observed for hydroxypropyl-β-CD. The patent also presents a comparative dissolution rate study of tablets containing the inclusion complex (SC and α-CD) and a physical mixture of the two compounds, indicating a slightly higher value for the IC. The same patent also demonstrates an increase in the SC concentration after oral administration of the IC when compared with a SC suspension [27].

The next report on inclusion complexes containing SC was made in 2013 by Sawatdee et al., who obtained three ICs with α-CD, HPBCD and γ-CD [24]. The purpose of the study was to obtain a nanosuspension of the IC for use in dose-metered inhalers. The choice of these cyclodextrins was supported by the fact that they were demonstrated to be safe for pulmonary use, HPBCD and γ-CD seeming to be the safest [28]. The phase solubility studies in distilled water indicated the highest increase in the solubility of SC for α-CD, followed by hydroxypropyl-β-CD and γ-CD, with the complexation constants varying in the same order. The results of the study showed in all cases that the dose-metered inhaler formulations containing the IC produced a drug content close to 100%, higher than the one obtained for the formulation containing only the active pharmaceutical ingredient (observed to be lower than 80%, with an average value of 31%) Matching results were later obtained by Atipairin et.al., who studied the development of a spray dried IC of SC and α-CD for use in dry-powder inhalers [29]. In 2017, Suwandecha et.al. demonstrated through spectrofluorometry that the IC containing β-CD was more stable than the one containing γ-CD [30].

In 2016, a study regarding an optimized orodissolvable film containing an inclusion complex of SC was published by Hosny et al. [23]. The study included three CDs: β-CD, hydroxypropyl-β-CD and hydroxybutyl-β-CD. The phase solubility studies indicated an increase in the solubility of SC as follows: nine times for the IC containing hydroxybutyl-β-CD, seven times for the IC containing β-CD and five times for the IC containing hydroxypropyl-β-CD. The values of the complexation constants follow the same order. For the orodispersible tablet, the IC with hydroxybutyl-β-CD was selected, and the results after oral administration indicated a 2.25-fold enhancement of the bioavailability of SC when compared to the marketed tablet.

The most recent study was published in 2024 by Sulistiawati et al. concerning the formation of an inclusion complex of SC with β-CD, which will be integrated in hydrogelforming microneedles for transdermal administration [25]. The study demonstrates that the complexation of SC significantly increases the solubility of the active molecule, facilitating its delivery through hydrogel-forming microneedles. This result is sustained by in vivo studies, along with the 5- to 14.6-fold increase in bioavailability when compared to oral administration. To ensure the maximal absorption of the active pharmaceutical ingredient, sodium starch glycolate was also added to the reservoir of the formulation.

### 1.2. Purpose of the Study

In this paper, our goal was to obtain and analyze the inclusion complex (IC) of SC and 2-hydroxypropyl-β-cyclodextrin (HPBCD), a cyclodextrin authorized by the FDA for inclusion in pharmaceutical formulations [31]. The next objective of this paper was the assessment of possible chemical interactions between the obtained IC and six pharmaceutical excipients (colloidal silicon dioxide, calcium lactate pentahydrate, α-lactose monohydrate, D-mannitol, polyvinylpyrrolidone K30 and methocel) at ambient temperature (via ATR-FTIR analysis coupled with statistical data analysis) and when heated under non-isothermal conditions (TG/DTG and DSC analysis) From the list of the selected excipients, three of them (α-lactose monohydrate, colloidal silicon dioxide and D-mannitol) have been proved to present possible chemical interactions with pure SC [32], and the compatibility of the other three (polyvinylpyrrolidone K30, methocel and calcium lactate pentahydrate) has not been evaluated. The six selected pharmaceutical excipients represent the most common excipients used in classical solid oral dosage formulations (tablets and capsules) By assessing the compatibility of the IC with those, various novel delivery systems could be developed, such as orodispersible, buccal and chewable tablets (colloidal silicon dioxide, calcium lactate pentahydrate, α-lactose monohydrate, D-mannitol and polyvinylpyrrolidone K30); dry-powder inhalers (α-lactose monohydrate) [29]; and transdermal systems (polyvinylpyrrolidone K30 and methocel).

The importance of compatibility studies resides in the necessity of assuring the optimal biopharmaceutical profile of the active pharmaceutical ingredient. The presence of chemical interactions between the active molecule and the pharmaceutical excipients can lead to degradation compounds which may present either a decrease in therapeutic effect or a potential toxic effect. The molecular mechanism underlying the chemical interactions between the active principle and excipients can be elucidated based on the functional groups present on each molecule, as well as environmental factors, such as temperature, typically by employing spectroscopic or chromatographic techniques. In both preformulation and formulation studies, demonstrating the occurrence of such induced interactions is often sufficient to restrict the use of certain excipients. These types of interactions can involve chemical transformations, such as esterification, hydrolysis, transesterification, Maillard reactions, condensations, dehydrations or oxidations, which are usually investigated separately in studies focused on drug impurity profiling [33]. The significance of these types of studies increases exponentially in the case of active pharmaceutical ingredients of which small doses are used, as with the use of SC in PAH, where one tablet contains 20 mg of the active principle, and where any degradation of the integrity of the active molecule may lead to therapeutic failure.

## 2. Materials and Methods

### 2.1. Molecular Modeling

The chemical structures of SC were acquired from the PubChem website (https://pubchem.ncbi.nlm.nih.gov) in SDF format, and to minimize the energy, the Gaussian 16 program suite (Wallingford, CT, USA) (DFT/B3LYP/6-311G level of theory) was used. The virtual structure of BCD (PDB ID: 6JEQ, X-ray diffraction, resolution 1.8 Å) was obtained from the Protein Data Bank in pdb file format. With the help of the initial structures of BCD, we drew HPBCD manually and optimized it in the same manner as SC. All the docking computations were performed in triplicate with the help of the Autodock 4.2.6 software (The Scripps Research Institute, San Diego, CA, USA) together with AutoDockTools. The docking between SC and HPBCD involves adding all the polar hydrogens and computing the Gasteiger charge; the grid box was created using Autogrid 4, with 60 × 60 × 60 Å in the x, y and z directions, with 0.375 Å spacing from the CD center. All the calculations were performed in a vacuum. To accomplish the docking process, we chose the Lamarckian Genetic Algorithm with a population size of 150 and 150 runs. All other parameters were used with the default values. To generate the molecular modeling figures, PyMol (http://www.pymol.org), The PyMOL Molecular Graphics System, Version 2.0 Schrödinger, LLC, New York, NY, USA, and Discovery Studio Visualization, Version 4.5 (Biovia) software were used.

### 2.2. Samples and Preparation

SC was according to European Pharmacopoeia Reference Standard and a commercial product of European Directorate for the Quality of Medicines and Healthcare EDQM, Council of Europe (batch 1.1, Id 010CY4, Strasbourg, France), the HPBCD cyclodextrin was obtained from Sigma-Aldrich (produced by Wacker Chemie AG, Burghausen, Germany), batch no. #BCBL4188V. The selected excipients for testing the pharmaceutical compatibility were colloidal silicon dioxide (SiO_2_, batch no.: 3157040314, Degussa AG., Essen, Germany), calcium lactate pentahydrate (CaL, batch no.: C8356, Sigma, Steinheim, Germany), α-lactose monohydrate (Lact·H_2_O, batch no.: #SLBK4809V, Sigma, Steinheim, Germany), D-mannitol (MNT, batch no.: #BCBM1675V, Sigma, Steinheim, Germany), polyvinylpyrrolidone K30 (PVP, batch no.: #BCBV7579, Sigma, Steinheim, Germany) and methocel (Met, #BCCB4283, Sigma, Steinheim, Germany) All the substances were used as received, without any further purification. The inclusion complex, with a molar ratio of 1:1 of SC and HPBCD, was prepared using the wet kneading method with absolute ethanol as the solvent. After the preparation, the IC was sieved and dried in a laboratory oven at the temperature of 40 °C for 48 h and then transferred in sealed brown vials. For testing of the compatibility of the obtained IC with the six selected pharmaceutical excipients, binary mixtures (BMs) were prepared with a mass ratio of 1:1 (IC:excipient) in an agate mortar and triturated in the presence of absolute ethanol until a constant mass was achieved (to guarantee the evaporation of the solvent, the samples were kept in a laboratory oven at 30 °C), after which they were placed in sealed brown vials and kept at 25 °C.

### 2.3. Stoichiometry and Stability Constant Determination

To establish the stoichiometry of the IC formed between SC and HPBCD, Job’s method was employed [34,35,36]. Two equimolecular solutions of SC and HPBCD were prepared, using distilled water as the solvent, at a concentration of 6.8 × 10^−5^ M. Afterwards, the solutions were mixed, varying the molar ratio of SC from 0.1 to 0.9. A similar set of solutions containing only the active pharmaceutical ingredient was prepared.

The Benesi–Hildebrand method was performed to confirm the stoichiometry of the IC and to determine the stability constant of the IC [37,38,39]. To achieve this, we measured the absorbance of a solution containing SC and HPBCD in excess. A solution of SC of 6.86 × 10^−5^ M was prepared (solvent: distilled water), and afterwards HPBCD was added in increasing concentrations from 0.343 × 10^−3^ M to 1 × 10^−3^ M (concentrations between 5 × [SC] and 100 × [SC]) To determine the stability constant of an IC with a stoichiometric ratio of 1:1, the following equation can be used (where Δε represents the change in the molar attenuation coefficient, ΔA is the change in absorbance and K is the stability constant of the IC):

(1) $$\frac{1}{∆A}=\frac{1}{∆\varepsilon \cdot \left[SC\right]\cdot K\cdot [HPBCD]}+\frac{1}{∆\varepsilon \cdot \left[SC\right]}$$

If the IC presents a stoichiometric ratio of 1:2, the stability constant can be determined with the following equation:

(2) $$\frac{1}{∆A}=\frac{1}{∆\varepsilon \cdot \left[SC\right]\cdot K\cdot {\left[HPBCD\right]}^{2}}+\frac{1}{∆\varepsilon \cdot \left[SC\right]}$$

The absorbance was determined using a Jena Analytik Specord 250 Plus double-beam spectrophotometer (Jena, Germany) Since SC presents two absorption maximum values in the UV domain (at 225 and 292 nm), the measurements were performed at the wavelength of 225 nm to complete the data presented in the scientific literature [23,24].

### 2.4. ATR–FTIR Investigations

To obtain the spectra of SC, HPBCD, IC and excipients, we used an IRSpirit Fourier Transform Infrared Spectrophotometer (Shimadzu, Kyoto, Japan) All the obtained spectra were built in the spectral range of 4000–400 cm^−1^ after 32 co-added scans and with a resolution of 2 cm^−1^. To perform the statistical calculations on the obtained data, the first step was the construction of a theoretical spectrum of the BMs with the help of the data of the pure compounds. The next step was to compare the theoretical spectrum with the experimental one with the help of Pearson’s correlation in the spectral regions, where significant absorption peaks were noticed for the IC or for the excipient. Accordingly, we did not include the 4000–3600 cm^−1^, 3200–3000 cm^−1^ (this region was kept for the BM containing Lact·H_2_O, MNT and CaL) and 2800–1800 cm^−1^ regions. To perform the statistical analysis, the MedCalc (version 23.1.3, MedCalc Software Ltd., Ostend, Belgium) software was used. If a correlation value between 1.00 and 0.80 was obtained, we were able to conclude the lack of chemical interactions between the components of the BM. A correlation value between 0.80 and 0.50 signifies that chemical interactions may occur in the BM at ambient temperature. With values lower than 0.50, the presence of chemical interactions at ambient temperature is confirmed [40,41,42,43].

### 2.5. Thermal Investigations

To acquire the TG/DTG data for SC, HPBCD, excipients and IC, a Netzsch TG 209 F1 Phoenix (Selb, Germany) instrument was used, with the sample placed in an open alumina crucible. The thermoanalytical curves were obtained under non-isothermal conditions at a heating rate of 10 °C min^−1^ from ambient temperature up to 450 °C in a dynamic air atmosphere with a flow rate of 20 mL min^−1^. The DSC curves were recorded at a heating rate of β = 10 °C min^−1^ in inert nitrogen medium with a flow rate of 20 mL min^−1^, from ambient temperature up to 250 °C, in sealed alumina crucibles, with the help of a Netzsch DSC 204 F1 Phoenix (Selb, Germany) instrument.

## 3. Results and Discussion

### 3.1. Molecular Modeling

AutoDock is a widely used molecular docking software package designed to predict how small molecules, such as drug candidates, bind to a target with a known 3D structure. AutoDock employs a combination of algorithms, including the Lamarckian Genetic Algorithm (LGA), to explore the possible orientations and positions of the ligand within the binding site. This approach enables it to efficiently search the conformational space of both the ligand and target, considering factors such as van der Waals forces, hydrogen bonding and electrostatic interactions. The software computes the binding affinity by calculating the free energy of binding, incorporating terms for intermolecular energy, torsional energy and desolvation effects. Its ability to model complex systems and provide insight into molecular interactions makes it a powerful tool for drug discovery and structural biology studies. Molecular modeling was employed to thoroughly characterize the interaction between SC and HPBCD in a 1:1 molar ratio. This approach enabled a detailed analysis of how sildenafil fits within the cyclodextrin cavity and the specific non-covalent interactions that stabilize the inclusion complex.

The docking studies revealed an estimated free binding energy of −6.20 kcal mol^−1^ for the most favorable pose of the SC/HPBCD complex, indicating a favorable and stable interaction between SC and HPBCD. The calculated inhibition constant (Ki) was 28.60 μM, which is consistent with a high binding affinity. A total of one hundred and fifty docking runs were performed, generating multiple binding poses. Although variability in ligand orientation within the cyclodextrin cavity was observed, the most energetically favorable pose was consistently selected based on binding free energy. Importantly, all major binding poses demonstrated similar patterns of interaction within the HPBCD binding site, supporting the reliability of the predicted binding mode.

**Figure 2:**
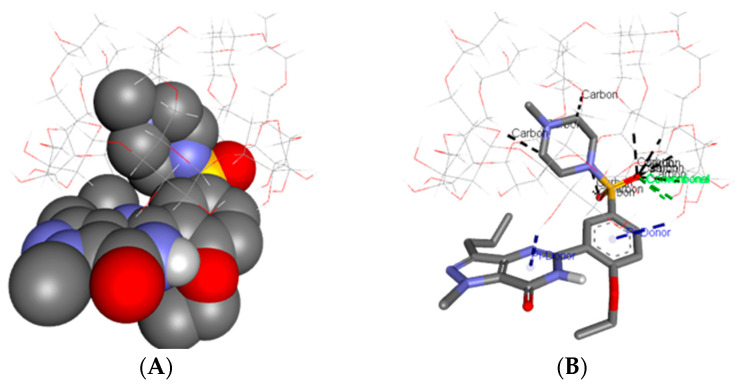

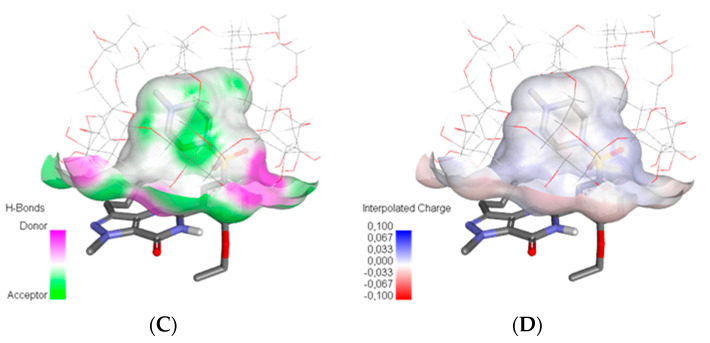
Inclusion complex simulation for a 1:1 molar ratio of SC and HPBCD. Image (**A**) shows the inclusion complex from the secondary face of the HPBCD cavity. SC guest molecules are represented by spheres and HPBCD is represented by sticks, both colored by elements. Image (**B**) shows polar/hydrophobic contacts between SC and HPBCD, SC being represented by sticks colored by elements and HPBCD being represented by lines. Image (**C**) shows the HPBCD surface colored by hydrogen bond type, with donors colored in green and acceptors in cyan, and Image (**D**) shows HPBCD surface colored by the interpolated atomic charges of the atoms.

The Figure 2 present theoretical models of the SC/HPBCD inclusion complex, as visualized using the Discovery Studio 4.5 molecular modeling environment and simulated at a 1:1 molar ratio.

In Figure 2A, the docking results clearly show that the polar sulfonyl-4-methylpiperazine moiety of sildenafil citrate (1-{[3-(1-methyl-7-oxo-3-propyl-6,7-dihydro-1H-pyrazolo [4,3-d]pyrimidin-5-yl)-4 ethoxyphenyl]sulfonyl}-4-methylpiperazine) is inserted into the hydrophobic cavity of HPBCD, with the encapsulation occurring from the secondary rim of the guest molecule.

Figure 2B demonstrates that the polar functional groups of SC, in particular the sulfonyl moiety, as well as the N-methylpiperazine ring, establish multiple classical and non-classical hydrogen bonds with the host molecule. Notably, the sulfonyl moiety acts as a principal hydrogen bond acceptor, establishing strong hydrogen bonds with the secondary hydroxyl functionalities of HPBCD, thereby contributing substantially to the stability of the inclusion complex. Additionally, the ethoxyphenyl and pyrazolo [4,3-d]pyrimidin-7-one moieties of sildenafil citrate are positioned in close proximity to the secondary cavity of HPBCD, enabling the formation of supplementary π-donor interactions with the cyclodextrin host, which may further stabilize the inclusion complex.

In the SC/HPBCD complex, thirteen non-classical hydrogen bonds were identified, including eleven C–H···O interactions (2.12 Å–3.58 Å) and two π-donor hydrogen bonds, with measured distances of 3.04 Å and 3.14 Å, respectively. These non-classical interactions indicate close spatial proximity and significant stabilization between the oxygen and nitrogen atoms of SC and the hydrogen atoms from the hydroxyl groups of HPBCD. Additionally, two classical hydrogen bonds, with bond lengths of 2.78 Å and 2.82 Å, were observed between the sulfonyl oxygen atom of SC and the hydrogen atoms of HPBCD. This pattern of interactions demonstrates that the SC molecule is anchored at the cyclodextrin rim via multiple hydrogen bonds, while its aromatic and hydrophobic moieties are favorably accommodated within the lipophilic cavity of HPBCD (Figure 2B) Figure 2C shows the molecular surface of the HPBCD in the SC/HPBCD inclusion complex, color-coded according to hydrogen bond donor (green) and acceptor (magenta) character. This visualization highlights the spatial distribution of polar functional groups at the host–guest interface. The HPBCD cavity entrance is characterized by a high density of hydrogen bond donor sites, primarily contributed by the hydroxyl functionalities of the cyclodextrin rim. These regions are positioned in close proximity to the polar acceptor sites on SC, most notably the sulfonyl and carbonyl oxygen atoms. In Figure 2D, the HPBCD cavity predominantly exhibits neutral to mildly negative potential, corresponding to the nonpolar carbohydrate backbone and hydroxypropyl substituents that comprise the hydrophobic interior. In contrast, pronounced regions of negative potential can be observed near the sulfonyl and carbonyl oxygen atoms of SC, particularly at the cavity entrance, where these groups are exposed toward the rim of HPBCD. These negatively charged sites are strategically positioned to engage in electrostatic and hydrogen bonding interactions with the cyclodextrin rim hydroxyl groups, which are capable of acting as hydrogen bond donors. The complementary distribution of electrostatic potential at the host–guest interface further supports the occurrence of favorable hydrogen bonding and electrostatic stabilization, as predicted by the docking analysis.

### 3.2. Stoichiometry and Stability Constant Determination

**Figure 3:**
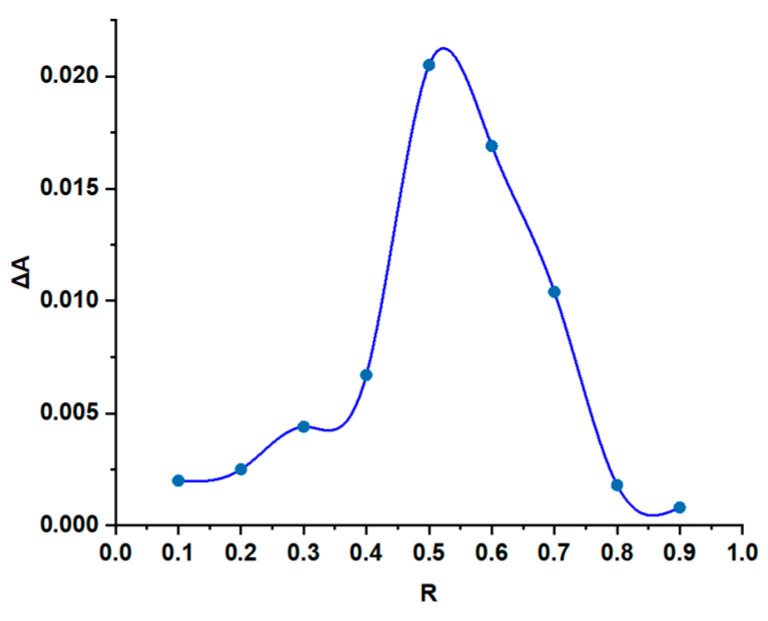
The results obtained via Job’s method for the IC between SC and HPBCD.

To determine the stoichiometry of the IC formed between SC and HPBCD with Job’s method, the value of the molar ratio at which the maximum absorbance value is obtained had to be identified. The graph shows the difference between the absorbance obtained for the solution containing SC + HPBCD minus the absorbance of the SC solution with the same molar ratio (ΔA(=A − A_0_) vs. the molar ratio (R)) In our study, the results obtained via Job’s method are presented in Figure 3, the molar ratio with the maximum absorbance value being at 0.5. This result suggests that the IC presents a 1:1 stoichiometry.

**Figure 4:**
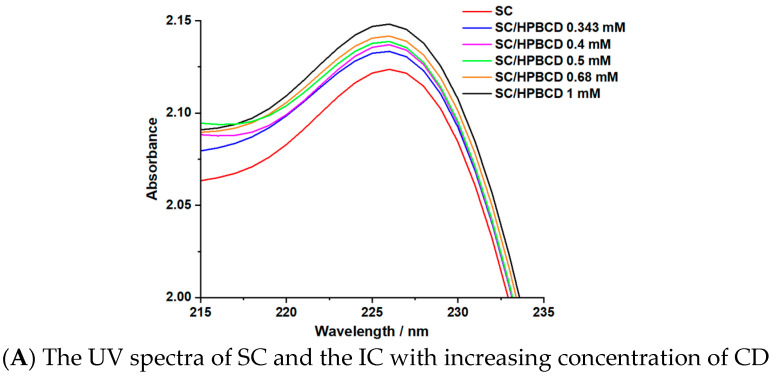

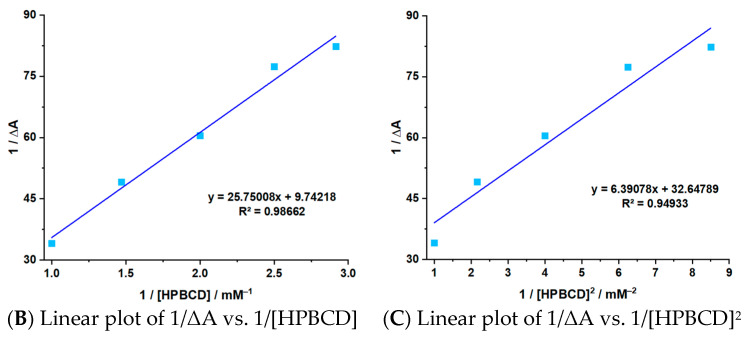
The results obtained via the Benesi–Hildebrand method.

To confirm the molar ratio of 1:1 in the IC, the Benesi–Hildebrand method was employed. If the IC respects a stoichiometry of 1:1, a linear fitting of 1/ΔA vs. 1/[HPBCD] should be obtained, where ΔA is the difference between the absorption of the IC and the absorption of SC at 225 nm. Otherwise, if a linear fitting of the 1/ΔA vs. 1/[HPBCD]^2^ is obtained, this means that the IC presents a stoichiometry of 1:2. Figure 4A represents the UV spectrum of SC and the IC in the presence of increasing concentrations of CD. In Figure 4B and 4C are presented the linear plotting of 1/ΔA vs. 1/[HPBCD] and 1/ΔA vs. 1/[HPBCD]^2^.

The results obtained via the Benesi–Hildebrand method confirm the results obtained via the molecular studies and Job’s method, namely, the obtainment of an IC with a molar ratio of 1:1 SC: HPBCD (the value of R^2^ for the linear plot of 1/ΔA vs. 1/[HPBCD] being greater than the one obtained from the linear plot of 1/ΔA vs. 1/[HPBCD]^2^) [44]. The stability constant for the obtained IC, determined at 225 nm, is equal to 378.3 M^−1^, a value in good agreement with the data presented in the scientific literature for the second absorption maximum of SC [23]. A stability constant value in the range of 100 and 5000 M^−1^ is considered optimal for the formation of the IC and indicates a favorable effect on the bioavailability of the active pharmaceutical ingredient [45,46,47,48].

### 3.3. ATR–FTIR Investigations

**Figure 5:**
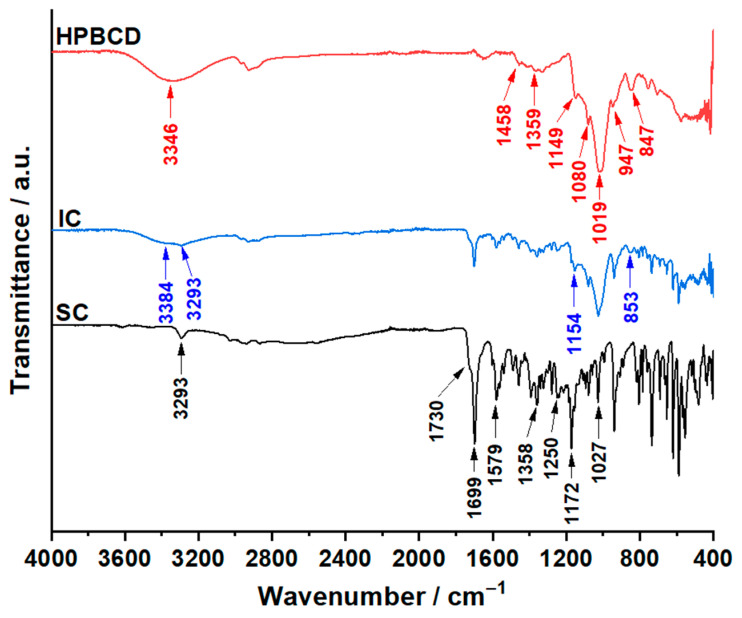
The ATR-FTIR spectra of SC, HPBCD and IC.

In Figure 5 and Table S1 are presented the results of the ATR-FTIR investigations employed for SC, HPBCD and the IC.

The FTIR spectra of SC revealed an absorption peak at 3293 cm^−1^ characteristic of the N-H moiety, observable at the pyrazolopyrimidine-7-one ring. In the spectral region of 3075−2800 cm^−1^, the stretching vibrations of the C-H moiety can be noticed at the following peaks: 3029, 2964, 2939, 2875 and 2864 cm^−1^. The intensification of this spectral region is caused by the hydroxyl groups present in citric acid. The stretching vibration of the carbonyl group from sildenafil and the carboxylic group of citric acid determines the appearance of the most intense absorption peak of the spectrum at 1699 cm^−1^, presenting a shoulder at 1730 cm^−1^. The symmetric stretching of the carboxyl radical is highlighted at 1579 cm^−1^. The asymmetric stretching vibrations of the sulfonamide group are represented as an intense absorption peak at 1358 cm^−1^, while the symmetrical ones are noticeable at 1172 cm^−1^. The C-O-C asymmetrical vibrations are revealed at 1250 cm^−1^ and the symmetrical vibrations at 1027 cm^−1^. The results of the ATR-FTIR investigation for the active pharmaceutical ingredient are in accordance with the data presented in the literature [49].

For the selected cyclodextrin, the first absorption peak that can be noticed on the spectrum is the intense absorption band with a peak at 3346 cm^−1^ belonging to the hydroxyl radicals. The stretching vibrations of the C-H moiety can be observed at 2968, 2925 and 2883 cm^−1^. The bending vibrations of the same moiety can be linked with the following absorption peaks: 1359 cm^−1^ (symmetrical) and 1458 cm^−1^ (asymmetrical) In the spectral region of 1190 and 879 cm^−1^ are highlighted the vibrations of the C-O moiety from the hydroxyl radical and the C-O-C moiety from the ether groups at 1149, 1080, 1019 and 947 cm^−1^.

For the IC, a few differences can be noticed when compared to the spectra of the pure compounds, SC and HPBCD. For the following absorption peaks, a shift can be noticed: 3384 → 3346 cm^−1^ (CD); 3033 → 3029 cm^−1^ (SC); 1154 → 1149 cm^−1^ (CD); and 853 → 847 cm^−1^ (CD) In addition, some peaks of SC merged with some of the HPBCD peaks: 2972 cm^−1^ (IC) is formed by 2964 cm^−1^ (SC) and 2968 cm^−1^ (CD), and 2931 cm^−1^ is formed by 2939 cm^−1^ (SC) and 2925 cm^−1^ (HPBCD) When comparing the spectra of the IC with the one of the HPBCD, the missing of the peaks from 2883, 1368, 1331, 1298 and 1019 cm^−1^ can be noticed. By comparing the spectra of the IC with the spectra of the active pharmaceutical ingredient, a large reduction in the intensity of the absorption bands for the sulfone group can be observed. Furthermore, at almost the same intensity as the pure molecule, the N–H moiety (3293 cm^−1^) and the symmetrical vibrations of the ether bond (1026 cm^−1^) are highlighted. Correlating the results of the ATR-FTIR investigations with the results of the molecular docking and the data presented in the literature (NMR analysis) [24], it can be concluded that the IC was formed and that the piperazine ring along with the sulfone group are entrapped in the cavity of HPBCD.

**Figure 6:**
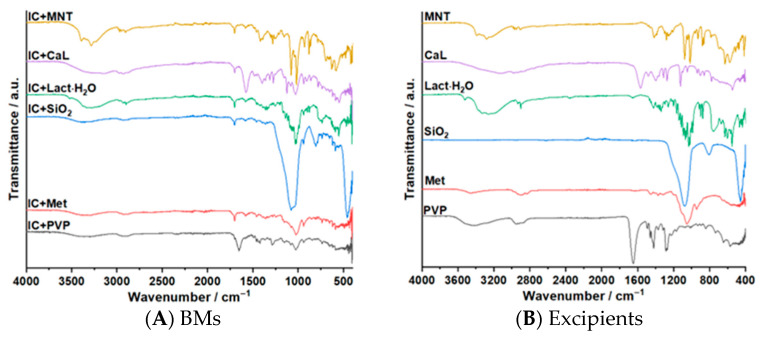

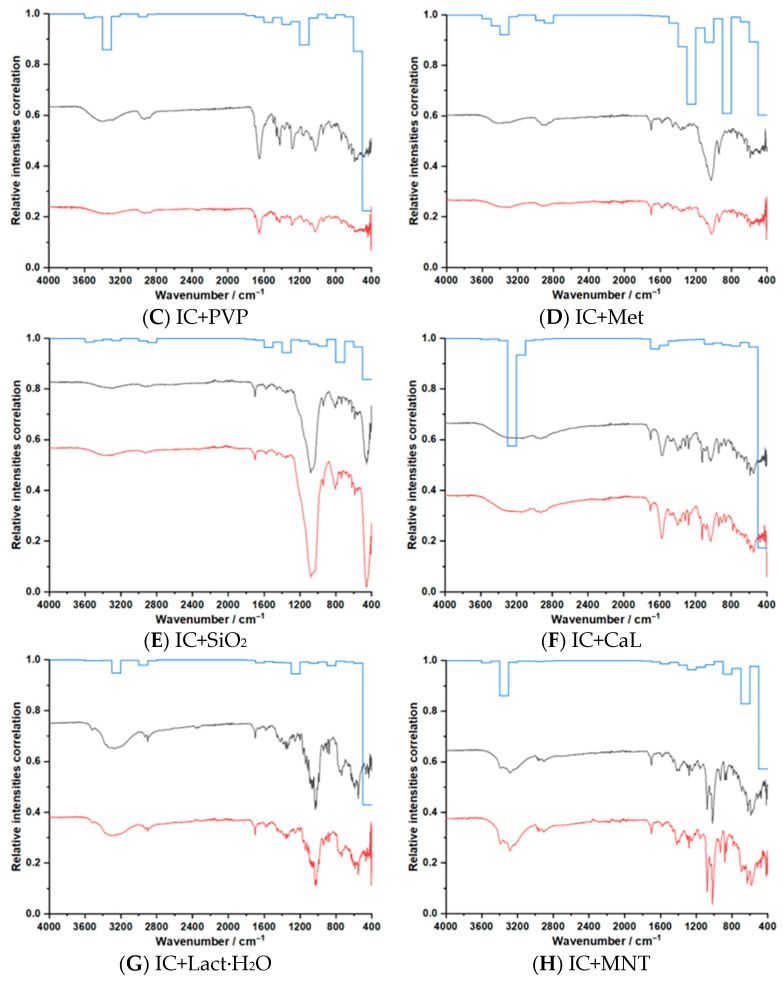
The spectra (practical with red and theoretical with black) and the Pearson’s correlation results obtained for each BM (**C**–**H**), the BMs (**A**) and the spectra of the excipients (**B**).

In Figure 6A–H, the ATR-FTIR investigations are presented along with the statistical results for each spectral region, and in Table S2 are shown the results of the ATR-FTIR analysis. To assess the absence of a chemical interaction between the investigated compounds and the pharmaceutical excipients at ambient temperature, their spectra need to be compared with those of the BMs, and no new absorption peaks or the absence of the main peaks of the pure compounds should be observed.

From the ATR-FTIR investigation and the Pearson’s correlation results for the BMs containing IC+MNT and IC+CaL, it can be concluded that at ambient temperature no chemical interactions between the complex and the selected pharmaceutical excipients occurs. Regarding the BMs with SiO_2_, PVP, Lact·H_2_O and Met, even if some changes can be observed in the spectral region of 3500−2900 cm^−1^, in the fingerprint region all the absorption bands corresponding to either the IC or the excipient can be found. Comparing these results with the statistical ones, where mainly values above 0.8 for the Pearson’s correlation were obtained, it can be concluded that at ambient temperature no chemical interactions between the IC and the four excipients are present.

### 3.4. Thermal Investigations

**Figure 7:**
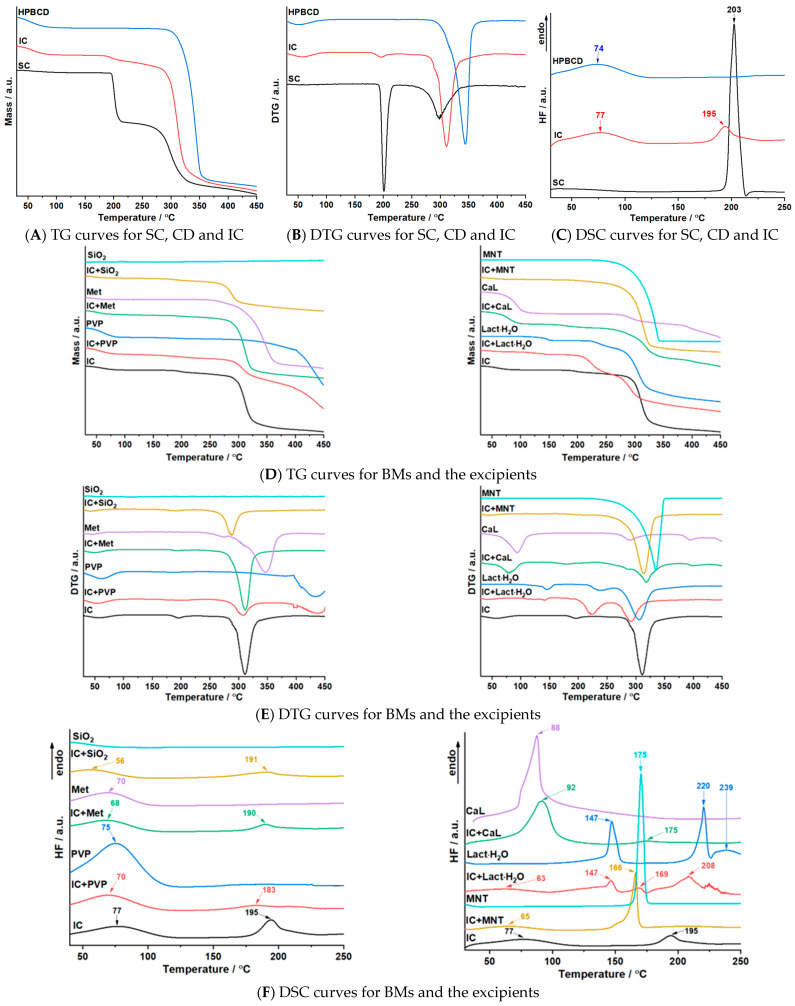
The thermoanalytical curves (TG/DTG and DSC) obtained for SC, HPBCD, IC and BMs.

The thermoanalytical curves TG/DTG and DSC obtained at a heating rate of β = 10 °C min^−1^ for the pure compounds; the IC and BMs are presented in Figure 7, while in Table 1 the interpretation of the graphs is highlighted.

The thermoanalytical profile of SC shows a thermal stability up to 188 °C and a degradation process consisting of two distinguishable processes. During the first one, which takes place in the temperature range of 188 to 220 °C (DTG maxima at 201 °C), SC loses about a quarter of its mass. The intense endothermic peak on the DSC curve at 203 °C represents the melting point of SC, a value in good agreement with the data presented in the literature [32]. The second degradation process begins at 243 °C and finishes at 353 °C, with a mass loss of 34.1%.

The first process on the DTG curve of HPBCD shows the dehydration of the cyclodextrin, which occurs in the temperature range of 30–100 °C, associated with the endothermic peak on the DSC curve at 74 °C. The degradation of the anhydrous cyclodextrin starts at 280 °C (DTG maximum at 344 °C) and involves the loss of more than three-quarters of the sample mass.

The decomposition process of the IC begins at an ambient temperature and finishes at 100 °C, with the maximum on the DTG curve at 57 °C, which can be associated with the loss of water from the cyclodextrin. This process is also highlighted by the DSC curve by an endothermic peak at 77 °C. The second degradation process of the IC, in the temperature range of 173–273 °C, belongs to the first decomposition process of the active pharmaceutical ingredient. The first aspect that illustrates the entrapment of SC in the selected cyclodextrin is represented by the shifting from 188 to 173 °C of the degradation process of the active substance. The third degradation process corresponds to the second degradation process of SC and the degradation of the anhydrous cyclodextrin. For this process a clear shift of the DTG maximum can be noticed at 311 °C, a value not observed on the thermoanalytical curves of the two components of the BM, which indicates the formation of the IC. Another aspect that demonstrates the entrapment of the active pharmaceutical ingredient in the cyclodextrin cavity is represented by the decrease in the melting point of SC from 203 to 195 °C.

The assertion of the existence of a chemical interaction between the IC and pharmaceutical excipients under thermal stress conditions is supported by the changes observed on the thermoanalytical curves, such as, in the case of TG/DTG, the change in the temperature at which the thermal degradation process begins, changes in the mass loss expected and the shifting of the DTG maximum temperature value, or, on the DSC curve the shifting of the endothermic or exothermic peaks, along with the reduction in their intensity. The mass loss expected is calculated as half of the mass percentage lost by the pure substance in the corresponding process.

The thermoanalytical curves of SiO_2_ do not indicate any significant process. The results of the thermal analysis for the BM of IC and SiO_2_ showed three decomposition processes. The first process is associated with the loss of water from the IC, the mass loss being equal to the expected one. In the case of the second process, it can be observed that it begins at a lower temperature, namely, 162 °C, but without a significant shift in the DTG maximum value. The DSC curve highlights in the same temperature range an endothermic peak at 191 °C, which also does not present a significant shift when compared to the one for the IC at 195 °C. The enthalpy obtained corresponds to the one expected (≈37 J g^−1^) The third degradation process occurs at a lower temperature than that of the IC, with a shift in the DTG maximum value being noted, the mass loss being equal to the one expected. These results show the lack of chemical interactions between the IC and the selected pharmaceutical excipient.

The TG/DTG curves of PVP revealed a decomposition consisting of two steps: the first process starts at 30 °C and finishes at 104 °C; the second one is in the temperature range of 312–450 °C. The first process corresponds to the loss of water, also highlighted on the DSC curve as an endothermic peak with a maximum at 75 °C. The second process corresponds to the degradation of anhydrous PVP. For the BM containing the IC and PVP, the results showed a four-step degradation process. The first one corresponds to the loss of water from the IC and the excipient. The second one corresponds to the second degradation process of the IC. The first thing to be noticed is the shift to a lower temperature, but the mass loss is equivalent to the expected one. For this process, the DSC curve revealed an endothermic peak at 183 °C. The third and fourth degradation processes are associated with the third decomposition process of the IC and the degradation of anhydrous PVP; Δm in both cases represents half of the loss of mass of the components of the BM in each process. Given the results, it can be concluded that no chemical interactions occur between the IC and the selected pharmaceutical excipient.

The thermoanalytical curves for CaL highlight the loss of water molecules during the first degradation process, followed by two processes associated with the decomposition of anhydrous calcium lactate. On the DSC curve an endothermic peak is revealed at 88 °C. Regarding the BM formed between the IC and the pharmaceutical excipient, a four-step decomposition process is noticeable. The first step presents the loss of water, followed by degradation of the IC from the BM, which begins at 143 °C, a much lower temperature than the one initially observed (173 °C) Other changes that can be noticed are represented by the shift of the DTG maxima from 195 °C to 179 °C and the shift of the endothermic peak on the DSC curve from 195 °C to 175 °C, with a strong reduction in intensity. Also, for the third degradation process, shifting to a lower temperature can be observed. The fourth degradation process is the only one starting at a higher temperature, 379 °C in comparison to 354 °C for the pure pharmaceutical excipient. In view of the obtained data, it can be concluded that under thermal stress a chemical interaction occurs between CaL and IC.

The degradation of Lact·H_2_O consists of three steps in the following temperature ranges: 95–167 °C, 213–265 °C and 265–410 °C, with a total loss of mass equal to 76.2%. For the BM, four degradation processes can be observed, the first two processes being represented by the loss of water from the IC (30–100 °C) and from the pharmaceutical excipient (100–155 °C) The third process corresponds to the second process of the IC and the second process of Lact·H_2_O and begins at 194 °C, a temperature between those of the two compounds and with a loss of mass higher than the one expected, 22.5% instead of 7.67%. The DSC curve revealed an endothermic peak at 169 °C, characteristic of the IC, but with a temperature decrease of 26 °C, along with an approximately 4-fold decrease in intensity. The fourth process corresponds to the third degradation processes of the IC and Lact·H_2_O, presenting a lower loss of mass than the one expected (≈64%), namely, 36.1%. Considering the results of the thermal analysis, it can be concluded that a chemical interaction occurs when the BM is heated.

The degradation of MNT consists of a one-step process in the temperature range of 221–352 °C, and the DSC curve revealed an intense endothermic peak at 175 °C, the melting point of the excipient, in accordance with the scientific data [50]. The thermoanalytical curves of the BM revealed a two-step degradation process. The first one corresponds to the water loss, while the second describes the degradation of the anhydrous IC and the excipient, starting at a higher temperature than the one of the IC. The DSC curve revealed only the endothermic peaks of dehydration, and the melting of MNT shifted to a lower value. From the results, it can be inferred that the IC chemical interacts with MNT when the BM is heated under non-isothermal conditions.

The thermoanalytical curves of Met revealed a degradation that occurs in three steps. The first one indicates the loss of water, highlighted on the DSC curve as an endothermic peak at 70 °C. The other two processes describe the decomposition of anhydrous Met. For the BM formed between the IC and Met a three-step degradation can be noticed. The first process, the loss of water from the IC and Met can be noticed, Δm being equal to the one expected. The second process is characteristic of the decomposition of the anhydrous IC, the loss mass and the start temperature being in accordance with the ones expected. The third process corresponds to the third decomposition noticed on the DTG curve of the IC and the two degradation processes of Met, the mass loss being equal to the expected one. On the DSC curve two intense endothermic peaks can be noticed, the first one at 68 °C characterizing the water loss and the second one at 190 °C corresponding to the melting of SC in the IC. These results demonstrate the lack of chemical interactions between the IC and Met when the BM is heated.

## 4. Conclusions

The first step of this study was the obtainment of an IC of SC and HPBCD in a molar ratio of 1:1, with the stoichiometry assessed according to the data presented in the scientific literature, and from the methods employed in this study: the molecular modeling, Job’s method and the Benesi–Hildebrand method. The stability constant determined with the data obtained for the first UV maxima for SC at 225 nm is equal to 378.3 M^−1^, a value in the range of 100 and 5000 M^−1^, which indicates a possible favorable effect for the bioavailability.

In the second part of the study, we tested the pharmaceutical compatibility of the obtained IC with six pharmaceutical excipients at ambient temperature (ATR–FTIR investigations) and under thermal stress (TG/DTG and DSC analysis); for three of them (Lact·H_2_O, SiO_2_ and MNT), the presence of possible chemical interactions with SC was stated in the literature, while for the other three the compatibility was not evaluated (PVP, Met and CaL) The results indicated the lack of chemical interactions at ambient temperature between the complex and the selected pharmaceutical excipients, a hypothesis confirmed by statistical analysis (Pearson’s correlation) When the BMs were exposed to thermal stress under non-isothermal conditions, for the ones containing Lact·H_2_O, CaL and MNT, chemical interactions were noticed, while the ones containing PVP, Met and SiO_2_ showed a lack of chemical interactions. Considering the results of the compatibility study, for the three excipients that did not show the presence of any chemical interactions at room temperature or when heated under non-isothermal conditions (PVP, Met and SiO_2_), no supplementary restrictions besides the ones recommended by the international guidelines should be considered. For the three BMs containing Lact·H_2_O, CaL and MNT, where the presence of chemical interactions was noticed under non-isothermal conditions between the inclusion complex and the excipient, precautions should be considered regarding the temperature variations during the manufacturing process, transport and storage.

## Figures and Tables

**Table 1:** The results of the thermal investigations.

Sample	Step	T_onset_ (°C)	T_offset_ (°C)	T_max DTG_ (°C)	T_peak DSC_ (°C)	ΔH_fus_ (J g^−1^)	**Δm (%)**
SC	I	188	220	201	203	380.2	27.4
	II	243	353	298			34.1
HPBCD	I	30	100	51	74	181.0	5.9
	II	280	374	344			84.4
SC+HPBCD (IC)	I	30	100	57	77 195	134.0 75.7	4.9
	II	173	273	195			6.2
	III	273	391	311			65.8
IC+ SiO_2_	I	30	100	42	56 191	82.1 36.0	2.7
	II	162	242	191			3.6
	III	242	334	288			29.1
SiO_2_	-	-	-	-	-	-	-
IC+PVP	I	30	100	54	70 183	156.2 42.4	7.7
	II	160	238	199			3.8
	III	253	352	309			26.4
	IV	352	450	437			34.4
PVP	I	30	104	63	75	409.2	11.6
	II	312	450	433			55.4
IC+CaL	I	30	117	80	92 175	499.4 10.5	15.3
	II	143	228	179			4.4
	III	228	373	286; 318			37.6
	IV	379	427	398			5.7
CaL	I	30	156	94	88	731.6	26.5
	II	244	354	288			12.0
	III	354	450	395			17.2
IC+Lact·H_2_O	I	30	100	43	63 147 169 208	54.6 85.8 17.0 109.7	2.3
	II	100	155	142			3.0
	III	194	256	224			22.5
	IV	256	344	292			36.1
Lact·H_2_O	I	95	167	144	147 220 239	150.0 153.3 43.6	5.0
	II	213	265	237			9.1
	III	265	410	305			62.1
IC+MNT	I	30	100	58	65 166	50.8 166.6	2.4
	II	240	345	314			79.5
MNT	I	221	352	312	175	295.0	97.1
IC+Met	I	30	100	48	68 190	118.3 28.6	5.0
	II	177	222	193			2.5
	III	264	358	311			66.9
Met	I	30	100	47	70	92.1	2.6
	II	232	282	275			5.3
	III	282	397	346			75.1

## Data Availability

Raw data are available upon request from the corresponding author of this work.

## Supplementary Materials

The following supporting information can be downloaded at: https://www.mdpi.com/article/10.3390/pharmaceutics17091114/s1, Table S1: The ATR-FTIR results for SC, HPBCD and IC; Table S2: The results of the ATR-FTIR investigation for the BMs.

## Abbreviations

The following abbreviations are used in this manuscript:

PAH	Pulmonary arterial hypertension
SC	Sildenafil citrate
HPBCD	2-Hydroxypropyl-beta-cyclodextrin
CD	Cyclodextrin
IC	Inclusion complex
BM	Binary mixture
SiO_2_	Aerosil
PVP	Polyvinylpyrrolidone K30
CaL	Calcium lactate pentahydrate
Met	Methocel
MNT	D-Mannitol
LGA	Lamarckian Genetic Algorithm
