# Supplementary material for: Compatibility Studies of Sildenafil-HPBCD Inclusion Complex with Pharmaceutical Excipients
Source: Pharmaceutics. 2025 Aug 27;17(9):1114. doi: 10.3390/pharmaceutics17091114 (PMC12473755; doi:10.3390/pharmaceutics17091114)
Supplement: Supplementary file 1 [file pharmaceutics-17-01114-s001.zip › pharmaceutics-3782335-supplementary.pdf]

Table S1. The ATR-FTIR results for SC, HPBCD and IC.

Sample	Spectral range (cm^{-1})		
	4000-1600	1600-1000	1000-650
**SC**	3293; 3029; 2964; 2939; 2875; 2864; 1730; 1699; 1603.	1579; 1563; 1540; 1490; 1459; 1392; 1358; 1342; 1324; 1301; 1278; 1250; 1240; 1215; 1185; 1172; 1108; 1095; 1080; 1059; 1027.	993; 939; 908; 891; 817; 805; 785; 759; 735; 691; 663; 653.
**HPBCD**	3346; 2968; 2925; 2883.	1457; 1368; 1331; 1298; 1149; 1080; 1019.	947; 847; 755; 704.
**IC**	3384; 3292; 3033; 2972; 2931; 2876; 1730; 1701; 1603.	1581; 1560; 1540; 1491; 1458; 1392; 1359; 1342; 1326; 1301; 1279; 1250; 1241; 1186; 1172; 1154; 1080; 1026.	940; 891; 853; 818; 805; 785; 759; 736; 691; 663; 654.

Table S2. The results of the ATR-FTIR investigation for the BMs.

Sample	Spectral range (cm^{-1})		
	4000-1600	1600-1000	1000-650
**IC+PVP**	**3408**; 3295; **2936**; 2871; 1701; **1654**.	1577; 1560; 1540; 1491; 1458; 1437; 1420; **1364**; 1341; 1319; 1288; 1223; 1172; 1154; 1081; 1027.	940; 844; 805; 785; 736; **669**; 653.
**PVP**	3417; 2950; 2921; 2885; 1648.	1493; 1461; 1435; 1421; 1372; 1317; 1286; 1272; 1227; 1169; 1073; 1017; 1001.	933; 896; 843; 733.
**IC+Met**	**3409**; **3297**; **2966**; 2931; 2873; 1733; 1700; 1604.	1582; 1560; 1540; 1490; 1458; 1394; 1361; 1341; 1326; 1280; 1251; 1242; 1172; 1153; 1080; 1024.	940; 891; 853; 818; 805; 785; 759; 736; 692; 653.
**Met**	3460; 2973; 2898; 2834.	1454; 1407; 1372; 1334; 1312; 1192; 1053.	944; 894; 657.
**IC+SiO_2**	**3355**; **2937**; **1723**; 1702.	1583; 1459; 1362; 1078; 1040.	941; 805; 759; 736; 691; 654.
**SiO_2**	–	1076.	809.
**IC+Lact·H_2O**	3522; **3374**; **3331**; **3267**; **3190**; 2976; 2934; 2900; 1730; 1701.	1582; 1560; 1540; 1457; **1437**; 1420; 1394; 1381; 1359; 1341; 1325; 1296; 1279; 1252; 1220; 1202; 1166; 1142; 1115; 1092;	988; 940; 915; 899; 876; 805; **778**; 758; 736; 654.

		1083; 1071; 1057; 1029; 1019; 1004.	
**Lact·H_2O**	3521; 3326; 3270; 2977; 2934; 2901.	1469; 1456; 1423; 1382; 1359; 1340; 1296; 1260; 1220; 1202; 1167; 1141; 1115; 1093; 1083; 1070; 1058; 1031; 1019; 1004.	988; 915; 899; 876; 757; 668.
**IC+CaL**	3293; **3142**; 2977; 2929; 2877; 1734; 1701.	1577; 1482; 1458; **1399**; 1362; 1313; 1275; 1253; 1171; 1154; 1124; 1081; 1028.	938; 904; 861; 818; 804; 780; 736; 691; 654.
**CaL**	3358; 3130; 2980; 2938; 2862; 2751; 2662.	1568; 1481; 1400; 1362; 1312; 1274; 1123; 1100; 1048.	937; 900; 861; 779; 682.
**IC+MNT**	3394; 3282; 3231; 2985; 2971; 2948; 2903; 1724; 1702.	1582; 1561; 1488; 1459; 1418; **1400**; 1389; **1314**; 1301; 1281; 1259; 1247; 1209; 1154; 1078; 1040; 1019.	958; 929; 881; 864; 805; 785; 759; 735; 692; 663; 653.
**MNT**	3391; 3283; 3230; 2985; 2971; 2947; 2903.	1484; 1460; 1417; 1388; 1355; 1300; 1281; 1259; 1246; 1232; 1209; 1078; 1045; 1018.	959; 929; 880; 863; 785; 762; 693; 658.
